# Design, Synthesis, Computational and Biological Evaluation of Novel Structure Fragments Based on Lithocholic Acid (LCA)

**DOI:** 10.3390/molecules28145332

**Published:** 2023-07-11

**Authors:** Jiangling Peng, Mingjie Fan, Kelly X. Huang, Lina A. Huang, Yangmeng Wang, Runkai Yin, Hanyi Zhao, Senlin Xu, Hongzhi Li, Alon Agua, Jun Xie, David A. Horne, Fouad Kandeel, Wendong Huang, Junfeng Li

**Affiliations:** 1 Department of Translational Research and Cellular Therapeutics, Arthur Riggs Diabetes and Metabolism Research Institute, Beckman Research Institute, City of Hope National Medical Center, Duarte, CA 91010, USA; 2 Department of Diabetes Complications & Metabolism, Arthur Riggs Diabetes and Metabolism Research Institute, Beckman Research Institute, City of Hope National Medical Center, Duarte, CA 91010, USA; 3 Department of Molecular Medicine, Beckman Research Institute, City of Hope National Medical Center, Duarte, CA 91010, USA

**Keywords:** bile acids, Takeda G protein-coupled receptor 5 (TGR5), metabolic disorders, hydrophilicity, luciferase-based reporter assay, structure–activity relationship (SAR)

## Abstract

The regulation of bile acid pathways has become a particularly promising therapeutic strategy for a variety of metabolic disorders, cancers, and diseases. However, the hydrophobicity of bile acids has been an obstacle to clinical efficacy due to off-target effects from rapid drug absorption. In this report, we explored a novel strategy to design new structure fragments based on lithocholic acid (LCA) with improved hydrophilicity by introducing a polar “oxygen atom” into the side chain of LCA, then (i) either retaining the carboxylic acid group or replacing the carboxylic acid group with (ii) a diol group or (iii) a vinyl group. These novel fragments were evaluated using luciferase-based reporter assays and the MTS assay. Compared to LCA, the result revealed that the two lead compounds **1a**–**1b** were well tolerated in vitro, maintaining similar potency and efficacy to LCA. The MTS assay results indicated that cell viability was not affected by dose dependence (under 25 µM). Additionally, computational model analysis demonstrated that compounds **1a**–**1b** formed more extensive hydrogen bond networks with Takeda G protein-coupled receptor 5 (TGR5) than LCA. This strategy displayed a potential approach to explore the development of novel endogenous bile acids fragments. Further evaluation on the biological activities of the two lead compounds is ongoing.

## 1. Introduction

Bile acids have long been recognized as detergents capable of solubilizing cholesterol and fatty acids to promote digestion and transport. Recent studies have revealed more expansive and profound paracrine and endocrine functions that have driven investigations on their therapeutic applications [1]. Drug development for metabolic disorders has harnessed the potential of bile acids as regulators of glucose and lipid metabolism, insulin sensitivity, and energy expenditure [2,3]. Downstream bile acid pathways have also exhibited a vast array of anti-inflammatory [4,5], cancer suppression [6,7], neuroprotection [8], viral inhibition [9], and intestinal and cardiovascular properties [10,11,12,13]. Therefore, the regulation of bile acid pathways is a promising therapeutic strategy for a host of metabolic disorders, cancers, and diseases. Due to their tremendous potential, the modulation of bile acids has arisen as an attractive approach for various therapies.

There has been corresponding growing interest in targeting the bile acid membrane receptors Takeda G protein-coupled receptor 5 (TGR5) and the farnesoid X receptor (FXR) [14]. TGR5 functions as a regulator of glucose metabolism, bile acid homeostasis, insulin secretion, and energy expenditure. As a membrane receptor, TGR5 is expressed in brown adipose tissue, skeletal muscle, the brain, the liver, the gallbladder, immune cells, and intestinal endocrine L-cells [15,16,17,18,19]. Meanwhile, FXR maintains energy and glucose homeostasis and liver metabolism. FXR is expressed primarily in the spleen, intestine, kidney, adrenal gland, and liver [20,21,22]. FXR activation and inhibition in the liver and small intestine is known to regulate downstream genes that reduce high-density lipoprotein (HDL) cholesterol activity, reduce obesity, lower serum triglycerides, and maintain bile acid homeostasis [23,24,25,26].

**Figure 1:**
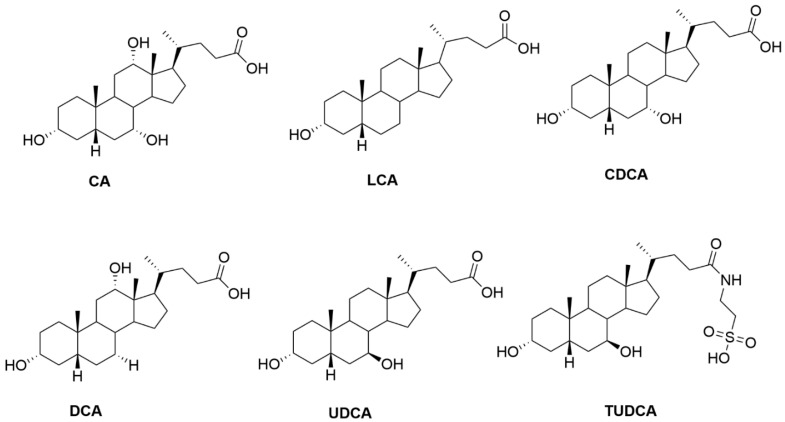
Chemical structures of endogenous bile acids that are TGR5 and/or FXR ligands.

Endogenous bile acids can generally be classified as hydrophobic, such as lithocholic acid (LCA) and deoxycholic acid (DCA), or hydrophilic, such as ursodeoxycholic acid (UDCA) and tauroursodeoxycholic acid (TUDCA) (Figure 1) [19,21,27,28,29]. The common primary bile acids, namely chenodeoxycholic acid (CDCA) and cholic acid (CA), are less hydrophobic than LCA and DCA but less hydrophilic than UDCA and TUDCA [30]. This classification underscores the importance of aqueous solubility in determining bile acid properties. In particular, the hydrophobic primary bile acids are initially toxic but are detoxified by liver enzymes to produce metabolites with higher water solubility [31]. The hydrophobic–hydrophilic balance of bile acids is crucial in bile acid homeostasis, as hydrophobic bile acids have been shown to induce inflammation, hepatocyte apoptosis, cytotoxicity, and cancer in gastrointestinal organs when exposed long term at high physiologic concentrations [32]. Hydrophilic bile acids, on the other hand, have been found to protect against cell death and exhibit anticancer effects, where prominence of effect depends on cell types [33,34,35]. Additional factors requiring consideration include solubility and membrane permeability, which are fundamental predictors of intestinal drug absorption and may be optimized to enhance the pharmacokinetic and clinical properties of synthetic agonists for drug discovery and development [36]. These factors are especially important in drug design since preclinical animal studies have revealed that systemic activation of bile acid pathways from rapid drug absorption results in off-target effects, such as pruritus and cardiovascular issues [37,38]. Increased doses of bile acids have also led to clinical cases of severe hepatotoxicity. These effects could be diminished by modulating intestinal absorption through hydroxylation to prevent bile acid accumulation in enterohepatic circulation [39]. As a result, improving bile acid hydrophilicity could reduce systemic side effects.

Despite the need to examine bile acid hydrophilicity, previous studies have primarily focused on modifying endogenous bile acids to improve potency and selectivity for TGR5 and/or FXR, rather than optimizing major fragments to improve hydrophilicity [40]. In this study, we reasoned that modification of the side chains of endogenous bile acids could enhance their hydrophilic properties to explore the development of new biological activity fragments of endogenous bile acids. The novel fragments were evaluated using luciferase-based reporter assays to elucidate their biological properties, and cell proliferation was determined by MTS assay. We applied our computational docking method to explore and explain the novel fragments–activity relationship. Considering the advantages of improved bile acid hydrophilicity, structurally modifying the side chain of bile acids may serve as an attractive strategy to design and optimize major fragments of endogenous bile acids.

## 2. Results and Discussion

### 2.1. A Novel Strategy to Design New Structure Fragments Based on LCA with Improved Hydrophilicity

**Figure 2:**
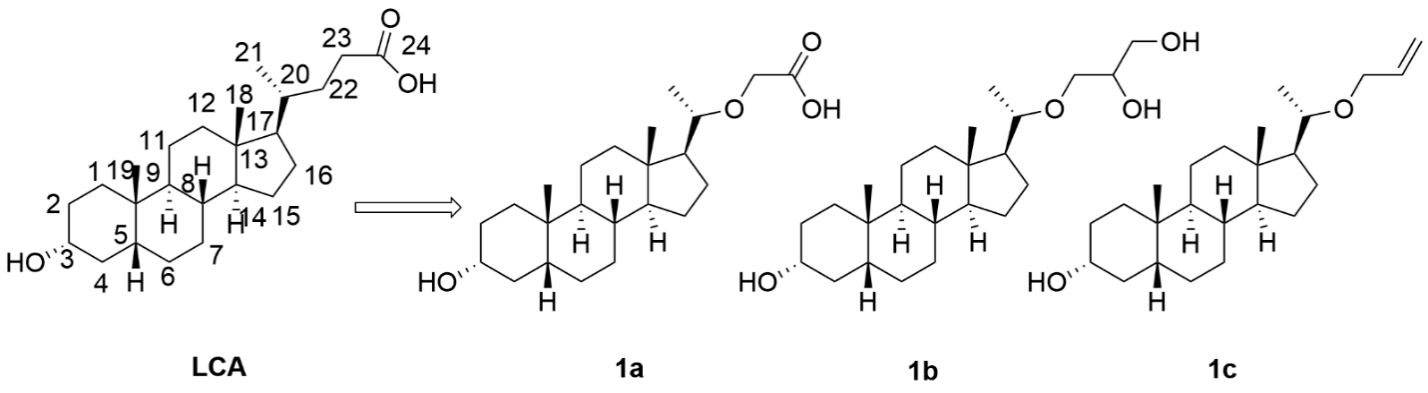
Strategy to design novel compounds **1a**–**1c** based on LCA.

Reports on modifying the side chains of endogenous bile acids for improving hydrophilicity are scarce. In fact, Wang et al. conducted the only modification study to date on 22-oxa-chenodeoxycholic acid (22-oxa-CDCA) [41]. However, they did not conduct any biological activity. Additionally, their synthesis method was limited. As introducing a polar “oxygen atom” has been an effective method to modify potent small molecule drugs, we hypothesized it would serve as an attractive starting point on the side chains for synthesizing novel bile acids. In the present study, we chose to design and optimize the major fragment of LCA, one of the most hydrophobic endogenous bile acids, by introducing a polar “oxygen atom” into the 22-position of the LCA side chain, then (i) either retaining the carboxylic acid group (**1a**) or replacing the carboxylic acid group with (ii) a diol group (**1b**) or (iii) a vinyl group (**1c**) (Figure 2). The logP values for compounds **1a**–**c** were calculated to be 3.93, 3.61, and 5.36, respectively. Compound **1a** (22-oxo-24-carboxylic acid-LCA) and compound **1b** (22-oxo-24,25-diol-LCA) demonstrated improved hydrophilic properties compared to LCA (3.93 and 3.61 vs. 5.30), while the logP value of compound **1c** (22-oxo-24-vinyl-LCA) was similar to that of LCA (5.36 vs. 5.30) as the control.

**Figure 3:**
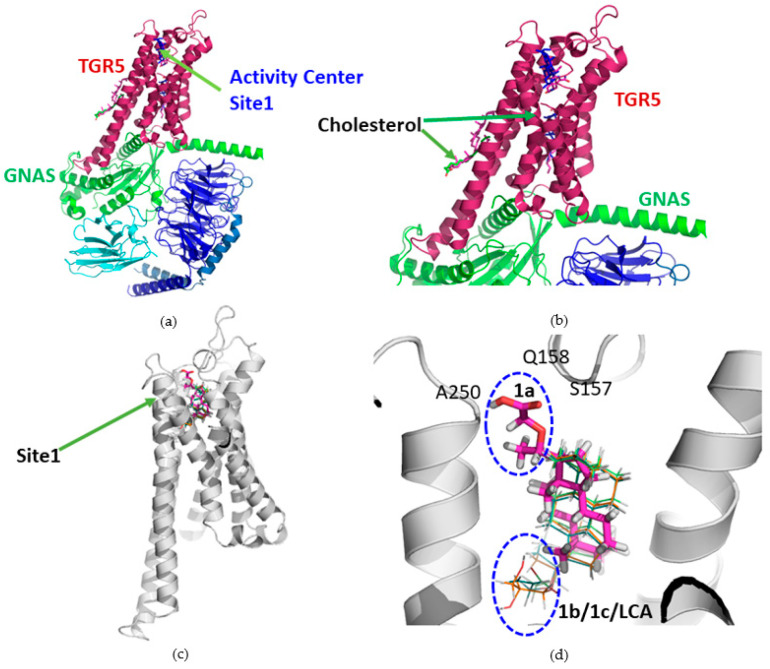
Activity center of TGR5 receptor and docking poses of LCA and its three analogs, **1a**–**1c**, in active center. (**a**) Protein TGR5 and its related activity center. (**b**) Magnification of TGR5 showing clear activity center. (**c**) LCA and its analogs **1a**–**1c** preferred to bind at the activity site of TGR5 protein. (**d**) Magnification of binding site of these analogs. Compounds **LCA**, **1b** and **1c** had very similar docking poses. Compound **1a** took an upside-down docking pose to form hydrogen bonds with S157, Q158, and A250 residues in TGR 5 protein.

Using our in-house-developed all-around docking method [42,43], we were able to predict the binding pocket and docking poses of LCA and its three analogs, **1a**–**1c**. According to Figure 3, LCA and its three analogs all preferred to bind at the activity site of the TGR5 protein with high docking scores, which is composed of residues of L71, L74, W75, Y89, P92, F96, S157, F161, Y240, T243, L244, S247, L266, and S270. Given the fairly high docking scores for all the compounds, syntheses of compounds **1a**–**1c** were performed consequently. However, a sub-pocket could also be formed by a specific network of hydrogen bonds between the side of bile acids and TGR5, which affected the compound’s activity, as discussed in Section 2.4. Structure–Activity Relationship Analysis.

### 2.2. Design and Synthesis of Compounds ***1a**–**1c***

Design and synthesis of compounds **1a**–**1c** began with commercially available pregnanediol. Because of the differences in the steric hindrance of the two hydroxy groups on pregnanediol, the 3-hydroxy group on pregnanediol could be selectively protected by *tert*-Butyldiphenylchlorosilane (TBDPSCl) to obtain compound **2** with a 59% yield.

**Scheme 1:**
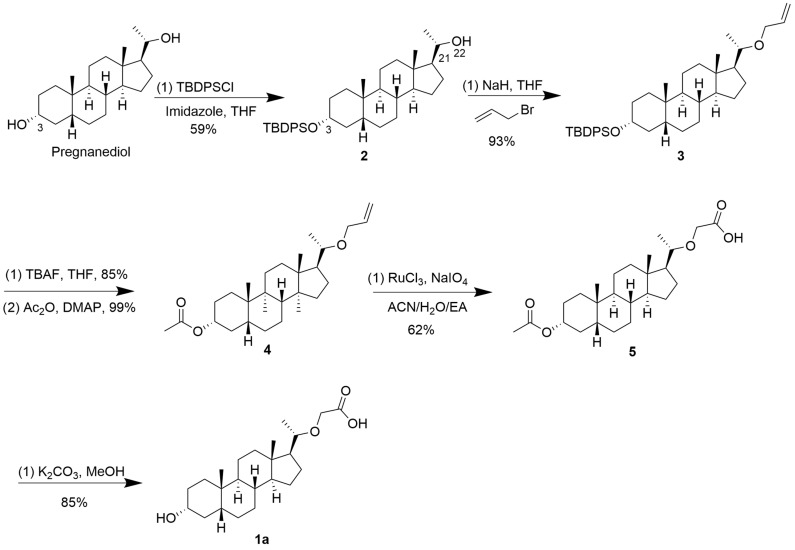
Synthesis route of compound **1a**.

The synthesis route for compound **1a** initially involved a 3-step synthetic route with compound **2** as the starting material: (i) reaction with ethyl bromoacetate (22-position); (ii) deprotection of TBDPS (3-position); and (iii) hydrolysis of the ester group (22-position). Unfortunately, there was no reaction between compound **2** and ethyl bromoacetate; we tested the reaction under multiple conditions, including different reaction temperatures, bases (potassium carbonate, sodium hydride or *t*-butyl lithium), and solvents (acetonitrile, DMF, DMSO, and THF). The failed reaction was determined to be potentially due to the large steric hindrance between the hydroxyl group in compound **2** and ethyl bromoacetate. This prompted us to modify the synthesis route (Scheme 1) using the allyl group, since this group could be easily transformed to a carboxyl acid group under oxidizing conditions. We experimented to develop a feasible synthesis method and compound **1a** was synthesized by inserting allyl group from compound **2**, deprotecting/protecting the 3-postion hydroxyl group to obtain compound **4**, oxidizing the vinyl group of compound **4** to a carboxyl group by means of RuCl_3_ as a catalyst and NaIO_4_ as an oxidant, and removing the acetate group of compound **5** in a solution of potassium carbonate in methanol.

**Scheme 2:**
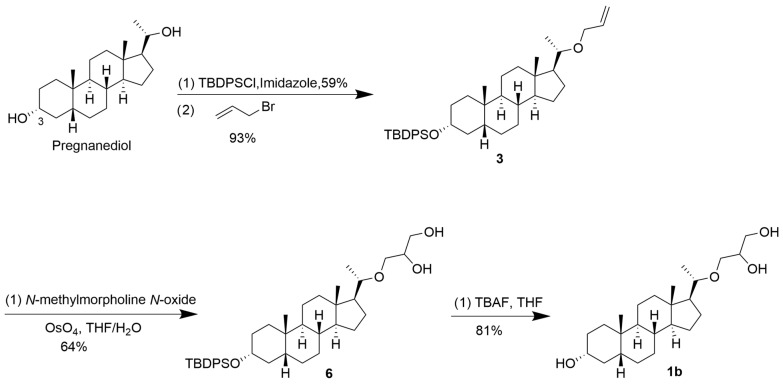
Synthesis route of compound **1b**.

Meanwhile, compound **1b** was designed from compound **3** (Scheme 2). For this procedure, we followed a method discovered by Hoffmann in 1912 to achieve *cis*-dihydroxylation of alkenes [44]. However, the key step of this method was the cycloaddition of osmium tetroxide (OsO_4_) to the olefin, necessitating the use of stoichiometric amounts of the toxic and the expensive reagent OsO_4_. To supplement this, Upjohn dihydroxylation was developed in 1976 and involves applying *N*-methylmorpholine *N*-oxide (NMO) as a stoichiometric re-oxidant for OsO_4_ [45]. Following Upjohn dihydroxylation, compound **3** was successfully oxidized to compound **6** without affecting the protected group TBDPS. Although ketone byproduct formation was reported for procedures following Upjohn dihydroxylation, we identified only a minute amount, which had no effect on purification, in the product. Finally, deprotection of TBDPS of compound **6** with TBAF afforded the target compound **1b** with 95% yield.

**Scheme 3:**
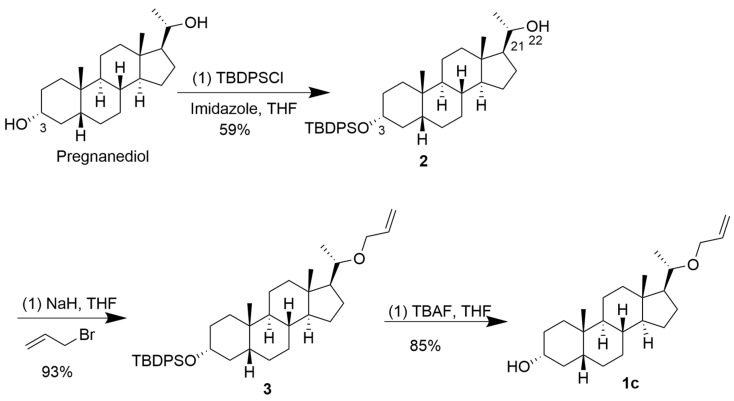
Synthesis route of compound **1c**.

The synthesis route for compound **1c** is shown in Scheme 3. Classic conditions of Ag_2_O and tetrabutylammonium iodide (TBAI) afforded compound **3**, but the reaction yield remained poor even when the reaction time was lengthened, and the temperature raised. The yield increased to 93% when we used sodium hydride as the base. Finally, deprotecting TBDPS with TBAF yielded the target compound **1c**.

### 2.3. Evaluated TGR5 Agonistic Activity and Cell Viability

**Figure 4:**
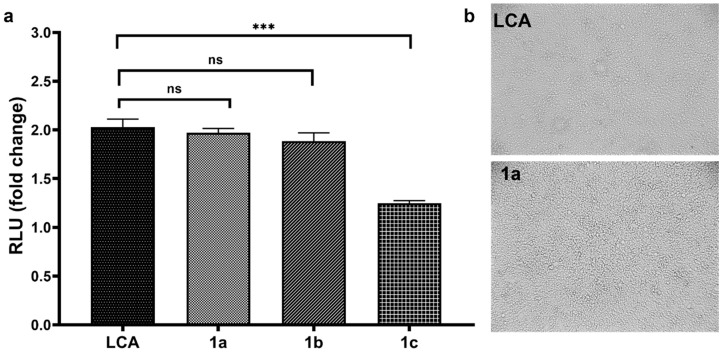
TGR5 agonistic activity of **LCA** and compounds **1a**–**1c** at 5 μM. (**a**) Data are presented as mean ± SE from *n* = 3 replicates. Bioactivity was compared between groups using unpaired *t*-tests. ns: no significance (*p* > 0.05); *** *p* < 0.001. (**b**) The representative images of HEK293T cells under treatment with 5 µM LCA and 5 µM compound **1a**. RLU, relative luciferase units.

To evaluate TGR5 agonistic activity, the novel compounds **1a**–**1c** and **LCA** were initially evaluated at concentrations of 5 μM via luciferase reporter assays based on our previous study to effectively activate TGR5 in vitro [40]. HEK293T cells were transfected with human TGR5 and cAMP-sensitive reporter plasmid pCRE-Luc and pCMV-Renilla as previously described [40]. At a low concentration, compound **1a** (1.97 ± 0.07) and compound **1b** (1.88 ± 0.15) exhibited agonist activity comparable to LCA (2.03 ± 0.14), whereas compound **1c** (1.25 ± 0.04) exhibited poor activity (Figure 4). The results showed that compounds **1a** and **1b** have no significantly different activity level than LCA, which indicated that the **1a** and **1b** active structure combined with TGR5 at the same active site and sub-pocket as predicted in pervious section.

We determined the changes in cAMP elicited by these LCA derivatives after binding to the TGR5 in order to determine their relative EC_50_ values. Compounds **1a** (EC_50_ = 4.688 µM) and **1b** (EC_50_ = 21.45 µM) displayed comparable activity to LCA (EC_50_ = 13.57 µM). However, **1c** had almost no improved potency compared to LCA. Compound **1a** had the lowest EC_50_ tested among the compounds, and two reasons might mainly be attributed to this. First, the lower EC_50_ of **1a** than LCA may be due to the improved hydrophilicity with the polar oxygen atom added. The improved hydrophilicity may result in prolonged absorption and a higher affinity to TGR5. This can prevent the bile acids from binding the uninterested organs. The efficiency increases as the hydrophilicity increases, yet more examinations still need to be performed to verify the hypothesis. Second, compound **1a** maintains the original carboxylic acid structure as LCA. It preserves the maximum similar structure as far as possible. The other two compounds changed the side chain attached to 22-position to a diol group and a vinyl group correspondingly. The change in the original structure may lead to a decrease in efficiency. Additionally, we observed a non-linear relationship when measuring the EC_50_ value for three compounds: the efficiency does not increase at a constant rate as the dosage increases. Other studies on GTR5 agonists also shared the common problem that might be due to the limited absorption [46,47].

In the present study, HEK 293T cells were treated by compounds **1a**–**c** and LCA concentrations from 0 to 25 µM. Cell viability was measured using the MTS assay. The findings revealed no statistically significant difference in cell viability across the range of 0–25 µM. This suggests that new bile acid structures **1a**–**1c**, under the 25 µM range, did not shown any sign of toxicity. The MTS assay results provided strong evidence that indicated that the increased activities, from pervious luciferase assay results, of compounds are independent of the toxicity within the specific 0 to 25 µM range. This study provides an encouraging outlook that the newly modified structures have a favorable safety profile with improved efficiency. However, in order to comprehensively analyze the implications of these results and ascertain the safety and effectiveness of the three compounds across multiple environments, further studies and evaluations are imperative.

### 2.4. Structure–Activity Relationship Analysis

Compounds **1a** and **1b** maintained similar potency and efficacy compared to LCA, whereas compound **1c** had poor activity. These results may be explained by previous reports [39,48], which showed that bile acids could bind to TGR5 with the carboxylic moiety making a hydrogen bond with the amino acid sequence in TGR5. Furthermore, a sub-pocket can be formed by a specific network of hydrogen bonds between the side of bile acids and TGR5. Compounds **1a** and **1b** were able to efficiently maintain the hydrogen bonds between their side chain and TGR5. However, a vinyl group on the side chain of compound **1c** disrupted the network of hydrogen bonds and therefore decreased TGR5 agonist activity. Thus, these bioactivity results indicated that increasing the hydrophilic properties of LCA to yield compounds **1a** and **1b** sustained binding activity.

**Figure 5:**
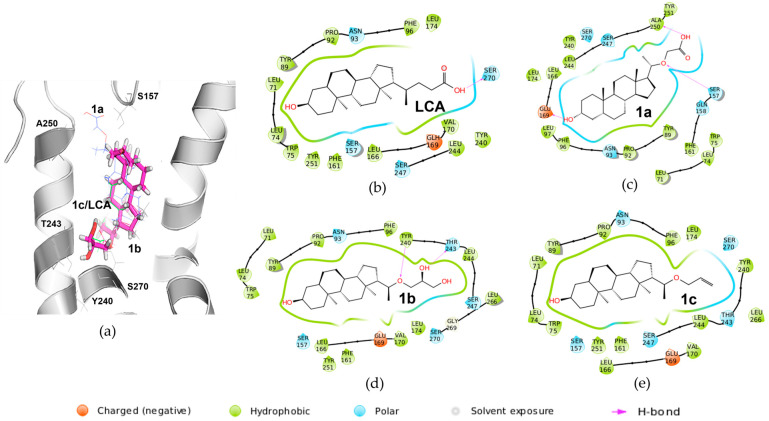
Explanation of bioactivities of **LCA** and its three analogs **1a**–**1c** using the binding pocket and docking poses within TGR5. (**a**) Docking poses of **LCA** and its analogs **1a**–**1c** overlapped together. (**b**) Two-dimensional interaction diagram of **LCA** with TGR5. **LCA** formed a hydrogen bond with S270 via hydrophilic acidic side (purple arrow). (**c**) Two-dimensional interaction diagram of **1a** with TGR5. Compound **1a** formed more hydrogen bonds with S157 and A250 residues in TGR5 protein compared to **LCA** (purple arrow). (**d**) Two-dimensional interaction diagram of **1b**. Compound **1b** formed more hydrogen bonds with Y240 and T243 in TGR5 protein compared to **LCA** (purple arrow). (**e**) Two-dimensional interaction diagram of **1c**. More hydrophobic compound **1c** could not form hydrogen bond with the hydrophilic residues anymore.

Further analysis of the binding pocket model of LCA and its analogs are shown in Figure 5. Panels for the interactions of these compounds with TGR5 depicted hydrophilic residues (S270/Y240/T243/A250/S57) that interacted with the hydrophilic ends of LCA and its analogs though the majority of the TGR5 activity center formed by the 7-helical bundle, which was hydrophobic. LCA formed a hydrogen bond with S270 via its hydrophilic acidic side, and analog **1a**/**1b** formed more hydrogen bonds with A250/S57 and Y240/T243 via their hydrophilic sides. However, more hydrophobic analog **1c** could not form a hydrogen bond with the hydrophilic residues anymore as demonstrated in Figure 5e. Thus, the decreasing agonistic activity of compound **1c** could be explained as follows: it lost hydrogen bond interaction with the hydrophilic residues in the activity center, which resulted in the lowest docking score of −8.12 kcal/mol and lowest binding affinity to block the activity center of TGR5.

Previous studies have developed different methods to modify non-bile-acid TGR5 agonists to optimize intestinal targeting and reduce systemic side effects. The incorporation of a large, polar group in synthetic TGR5 agonists by Lasalle et al. reduced intestinal absorption and increased intestine-specific oral drug delivery [49]. The lead compound improved glucose tolerance in a murine model of obesity and insulin resistance. Another study incorporated polar functional groups to prepare a TGR5 agonist with low absorbance and decreased off-target activation of the gallbladder [50]. Furthermore, a potent gut-restricted TGR5 agonist demonstrated minimal gallbladder-related side effects in mice [51]. OL3, another TGR5 agonist with a hydrophilic side chain, effectively lowered blood glucose without gallbladder filling [52]. However, these studies have focused on non-bile-acid-derived synthetic molecules. In this study, we report the development of compounds **1a** and **1b** from the endogenous bile acid LCA through a novel methodology of introducing polar groups containing an oxygen base and retaining or modifying the carboxylic acid group. It was reported that increasing hydrophilicity could potentially limit intestinal permeability and reduce the side effects of LCA and its derivatives, which is particularly beneficial given that LCA is the most hydrophobic endogenous bile acid [53]. Furthermore, bile acids and derivatives beyond LCA may also benefit from a high topological polar surface due to the reduction in adverse effects from off-target binding [54], such as obeticholic acid (OCA), CDCA, and TUDCA, which have relatively hydrophobic bile acids. Current modifications of these compounds do not focus on their side chain. With the strategy we developed to obtain new bile acid analogs with improved hydrophilicity and comparable binding activity (**1a** and **1b**), bile acids demonstrating clinical potential could conceivably undergo similar permeability modifications to potentially reduce systemic activation and clinical side effects.

## 3. Materials and Methods

All reagents and chemicals were purchased from commercial suppliers and used without further purification unless otherwise stated. ^1^H and ^13^C spectra were recorded at Bruker 700 and 175 MHz, respectively, and the chemical shifts for ^1^H-NMR and ^13^C-NMR were referenced as tetramethylsilane (TMS) via residual solvent signals. The resonance patterns are indicated as follows: s, singlet; d, doublet; t, triplet; q, quartet; m, multiplet; dd, doublet of doublets; and dt, doublet of triplets. The coupling constants (*J*) are reported in Hz. Mass spectra were obtained on an Agilent lnfinitylab mass spectrometer equipped with an ESI source operated in positive mode or negative mode. Flash column chromatography was preformed over silica gel of 200–300 mesh. 

### 3.1. Chemistry

*(1S)-1-((3R,10S,13S)-3-((Tert-butyldiphenylsilyl)oxy)-10,13-dimethylhexadecahydro-1H-cyclopenta[α]phenanthren-17-yl)ethan-1-ol* (**2**). To a solution of (3*R*,10*S*,13*S*)-17-((*S*)-1-hydroxyethyl)-10,13-dimethylhexadecahydro-1*H*-cyclopenta[α]phenanthren-3-ol (Pregnanediol) (500 mg, 1.6 mmol, 1 Eq) and imidazole (325 mg, 3 Eq) in DCM (100 mL) at room temperature under nitrogen atmosphere, *tert*-Butyl(chloro)diphenylsilane (650 mg, 1.2 Eq) was added. After 1 h, the reaction mixture was diluted with water (100 mL). The organic phase was separated, and the aqueous phase was extracted with DCM (3 × 100 mL). The organic phases were then combined and washed with water (3 × 100 mL) and brine (2 × 100 mL) and dried with sodium sulfate. The organic solvent was removed under reduced pressure. The crude product 2 was purified by silica gel flash column chromatography with DCM elution to furnish compound 2 as a sticky oil (525 mg, 59% yield). ^1^H-NMR (700 MHz, CDCl_3_): δ 7.67–7.69 (m, 4H), 7.40–7.42 (m, 2H), 7.35–7.38 (m, 4H), 3.64–3.40 (m, 1H), 3.60–3.63 (m, 1H), 1.81–1.92 (m, 3H), 1.7–1.8 (m, 1H), 1.45–1.71 (m, 5H), 1.32–1.46 (m, 6H), 1.12–1.36 (m, 10H), 1.05 (s, 9H), 0.82 (s, 3H), 0.71–0.76 (m, 1H), 0.63 (s, 3H).
*(((3R,10S,13S)-17-((S)-1-(allyloxy)ethyl)-10,13-dimethylhexadecahydro-1H-cyclopenta[α]phenanthren-3-yl)oxy)(tert-butyl)diphenylsilane* (**3**). To a suspension of sodium hydride (NaH) in THF (4 mL) in a sealed tube at room temperature compound 2 (350 mg, 0.63 mmol) in THF (2 mL) was added dropwise. The mixture was stirred at room temperature for 30 min. Then, allyl bromide (150 mg, 2 Eq) was added to the above reaction mixture. The reaction mixture was stirred and heated at 110 °C for 8 h. The reaction was quenched with water (20 mL) at room temperature. The reaction mixture was then extracted with ethyl acetate (3 × 20 mL), dried over sodium sulfate, and filtered and concentrated under reduced pressure. The crude product was purified by silica gel flash column chromatography with hexane and ethyl acetate (*v*/*v*, 50:1) as eluents to afford compound 3 as a white solid (350 mg, 93% yield). ^1^H-NMR (700 MHz, CDCl_3_): *δ* 7.67–7.69 (m, 4H), 7.40–7.42 (m, 2H), 7.35–7.38 (m, 4H), 5.89–5.94 (m, 1H), 5.25 (d, *J* = 16.8 Hz, 1H), 5.12 (d, *J* = 10.5 Hz, 1H), 4.06–4.09 (m, 1H), 3.80–3.84 (m, 1H), 3.60–3.63 (m, 1H), 3.28–3.63 (m, 1H), 1.91–1.98 (m, 1H), 1.82–1.86 (m, 2H), 1.72–1.76 (m, 2H), 1.31–1.55 (m, 13H), 1.16 (d, *J* = 6.3 Hz, 3H), 1.09–1.15 (m, 3H), 1.05 (s, 9H), 0.81 (s, 3H), 0.73 (m, 1H), 0.61 (s, 3H).
*(3R,10S,13S)-17-((S)-1-(Allyloxy)ethyl)-10,13-dimethylhexadecahydro-1H-cyclopenta[α]phenanthren-3-ol* (**1c**). To a solution of compound **3** (350 mg, 0.59 mmol) in THF (3 mL) at room temperature, TBAF was added (3 mL, 1 M, in THF). The resulting mixture was stirred at room temperature overnight. The solvent was then removed under reduced pressure, and the residue was purified by silica gel flash column chromatography with hexane and ethyl acetate as eluents (*v*/*v*, 5:1) to furnish compound **1c** as a white solid (180 mg, 85% yield). ^1^H-NMR (700 MHz, CDCl_3_): δ 5.87–5.93 (m, 1H), 5.24 (d, *J* = 16.8 Hz, 1H), 5.10 (d, *J* = 10.5 Hz, 1H), 4.05–4.08 (m, 1H), 3.80–3.83 (m, 1H), 3.60–3.63 (m, 1H), 3.27–3.30, (m, 1H), 1.91–1.96 (m, 1H), 1.81–1.86 (m, 2H), 1.73–1.79 (m, 2H), 1.65–1.69 (m, 1H), 1.46–1.54 (m, 2H), 1.34–1.43 (m, 7H), 1.17–1.34 (m, 3H), 1.15 (d, *J* = 6.3 Hz, 3H), 1.04–1.13 (m, 4H), 0.96–0.99 (m, 1H), 0.91 (s, 3H), 0.62 (s, 3H). ^13^C-NMR (175 MHz, CDCl_3_), δ 136.25, 116.60, 78.24, 72.36, 69.79, 57.63, 56.86, 42.60, 40.99, 39.82, 36.91, 36.06, 35.87, 31.02, 30.99, 27.67, 26.93, 26.90, 26.86, 24.61, 23.85, 21.07, 21.04, 19.69, 13.10. MS for C_24_H_40_O_2_ calculated: *m*/*z* 383.3 (M + Na)^+^; found: 383.3.
*(3R,10S,13S)-17-((S)-1-(Allyloxy)ethyl)-10,13-dimethylhexadecahydro-1H-cyclopenta[α]phenanthren-3-yl acetate* (**4**). To a solution of (*3R*,*10S*,*13S*)-17-((*S)*-1-(allyloxy)ethyl)-10,13-dimethylhexadecahydro-1*H*-cyclopenta[α]phenanthren-3-ol, **1c** (180 mg, 0.5 mmol) and 4-Dimethylaminopyridine (DMAP) (132 mg, 1.5 Eq) in dichloromethane (DCM) (6 mL) at room temperature, acetic anhydride (60 mg, 1.2 Eq) was added. The resulting mixture was stirred at room temperature for 4 h. The solvent was then removed under reduced pressure and the residue was purified by silica gel flash column chromatography with hexane and ethyl acetate as elution (*v*/*v*, 20:1) to furnish compound **4** as a viscous oil (200 mg, 99% yield). ^1^H-NMR (700 MHz, CDCl_3_): δ 5.85–5.89 (m, 1H), 5.23 (d, *J* = 16.8 Hz, 1H), 5.09 (d, *J* = 10.5 Hz, 1H), 4.67–4.71 (m, 1H), 4.03–4.06 (m, 1H), 3.78–3.81 (m, 1H), 3.24–3.28 (m, 1H), 2.0 (s, 3H), 1.89–1.94 (m, 1H), 1.77–1.86 (m, 4H), 1.64–1.67 (m, 2H), 1.55–1.59 (m, 1H), 1.45–1.54 (m, 2H), 1.34–1.43 (m, 8H), 1.17–1.23 (m, 2H), 1.13 (d, *J* = 6.3 Hz, 3H), 1.02–1.08 (m, 5H), 0.96–1.02 (m, 1H), 0.90 (s, 3H), 0.60 (s, 3H).
*2-((1S)-1-((3R,10S,13S)-3-Acetoxy-10,13-dimethylhexadecahydro-1H-cyclopenta[α]phenanthren-17-yl) ethoxy) acetic acid* (**5**). Compound **4** (200 mg, 0.5 mmol) and RuCl_3_ (10 mg, 0.1 Eq) were dissolved in a mixed solvent of acetonitrile (4 mL), water (6 mL) and ethyl acetate (4 mL) at room temperature, and then NaIO_4_ (560 mg, 4 Eq) was added. The resulting mixture was stirred at room temperature overnight. The reaction mixture was then diluted with water (60 mL) and ethyl acetate (60 mL). The aqueous phase was separated and extracted with ethyl acetate (3 × 60 mL). The organic phases were combined and washed with water (2 × 30 mL) and brine (2 × 30 mL), followed by drying using sodium sulfate. The solvents were removed under reduced pressure and the residue was purified by silica gel flash column chromatography with dichloromethane and methanol as elution (*v*/*v*, 25:1) to furnish compound **5** as a viscous oil (130 mg, 62% yield). ^1^H-NMR (700 MHz, CDCl_3_): δ 4.58–4.61 (m, 1H), 4.11 (d, *J* = 9.1 Hz, 1H), 3.93 (d, *J* = 16.1 Hz, 1H), 3.40–3.45 (m, 1H), 2.0 (s, 3H), 1.89–1.94 (m, 1H), 1.77–1.86 (m, 4H), 1.61–1.69 (m, 2H), 1.35–1.56 (m, 9H), 1.21–1.27 (m, 2H), 1.19 (d, *J* = 6.3 Hz, 3H), 1.06–1.17 (m, 4H), 0.96–1.02 (m, 1H), 0.91 (s, 3H), 0.62 (s, 3H).
*2-((1S)-1-((3R,10S,13S)-3-Hydroxy-10,13-dimethylhexadecahydro-1H-cyclopenta[α]phenanthren-17-yl) ethoxy) acetic acid* (**1a**). To a solution of compound **5** (130 mg, 0.31 mmol) in MeOH (10 mL) at room temperature K_2_CO_3_ (1.07 g in 0.5 mL water, 25 Eq) was added aq. The resulting mixture was stirred for 4 h. The reaction mixture was then quenched with water (50 mL) and adjusted to a pH of 2 with 3M hydrochloric acid. The white precipitate was extracted with ethyl acetate (3 × 50 mL). The organic phases from each of the extractions were combined and dried with sodium sulfate. The solvent was removed under reduced pressure, and the residue was purified by silica gel flash column chromatography with dichloromethane and methanol elution (*v*/*v*, 25:1) to produce compound **1a** as a white solid (100 mg, 85% yield). ^1^H-NMR (700 MHz, CDCl_3_): δ 4.12 (d, *J* = 16.1 Hz, 1H), 3.92 (d, *J* = 16.1 Hz, 1H), 3.59–3.64 (m, 1H), 3.42–3.46 (m, 1H), 1.89–1.94 (m, 1H), 1.77–1.86 (m, 2H), 1.71–1.79 (m, 2H), 1.63–1.66 (m, 3H), 1.45–1.61 (m, 3H), 1.35–1.43 (m, 6H), 1.22–1.32 (4H), 1.06–1.23 (m, 7H), 0.94–0.98 (m, 1H), 0.90 (s, 3H), 0.62 (s, 3H). ^13^C-NMR (175 MHz, CDCl_3_): δ 80.94, 72.34, 65.90, 57.06, 56.77, 42.51, 40.91, 39.71, 36.84, 36.00, 35.80, 30.94, 27.59, 26.93, 26.87, 24.56, 23.81, 21.02, 19.53, 13.12. MS for C_23_H_38_O_4_ calculated: *m*/*z* 377.3 (M − H)^−^; found: 377.3.
*3-((1S)-1-((3R,10S,13S)-3-((Tert-butyldiphenylsilyl) oxy)-10,13-dimethylhexadecahydro-1H-cyclopenta[α]phenanthren-17-yl) ethoxy)propane-1,2-diol* (**6**). (((3*R*,10*S*,13*S*)-17-((*S*)-1-(allyloxy) ethyl)-10,13-dimethylhexadecahydro-1*H*-cyclopenta[α]phenanthren-3-yl) oxy)(*tert*-butyl)diphenylsilane, 3 (200 mg, 0.34 mmol, 1 Eq) and OsO_4_ (4.5 mg, 0.05 Eq) were dissolved in a mixture solvent of THF (1 mL) and water (1 mL), and then *N*-methylmorpholine *N*-oxide (80 mg, 0.68 mmol, 2 Eq) was added at room temperature. The resulting mixture was stirred at room temperature overnight. The reaction mixture was then diluted with water (30 mL) and ethyl acetate (30 mL). The aqueous phase was separated and extracted with ethyl acetate (3 × 30 mL). The organic phases were combined and washed with water (2 × 30 mL) and brine (2 × 30 mL), followed by drying with sodium sulfate. The solvent was removed under reduced pressure, and the residue was purified by silica gel flash column chromatography with dichloromethane and methanol as elution (20:1) to furnish compound 6 as a viscous oil (138 mg, 64% yield). ^1^H-NMR (700 MHz, CDCl_3_): δ 7.65–7.67 (m, 4H), 7.37–7.41 (m, 2H), 7.33–7.35 (m, 4H), 3.77–3.79 (m, 1H), 3.67–3.72 (m, 1H), 3.55–3.64 (m, 2H), 3.35–3.42 (m, 1H), 3.27–3.31 (m, 1H), 3.24–3.26 (m, 1H), 2.58 (dd, *J* = 19.6, 5.6 Hz, 1H), 2.19 (d, *J* = 19.6 Hz, 1H), 1.81–1.90 (m, 2H), 1.71–1.79 (m, 2H), 1.68–1.71 (m, 1H), 1.55–1.61 (m, 5H), 1.35–1.51 (m, 7H), 1.12–1.21 (m, 8H), 1.03 (s, 9H), 0.79 (s, 3H), 0.58–0.62 (m, 1H), 0.59 (s, 3H).
*3-((1S)-1-((3R,10S,13S)-3-Hydroxy-10,13-dimethylhexadecahydro-1H-cyclopenta[α]phenanthren-17-yl)ethoxy)propane-1,2-diol* (**1b**). To a solution of compound 6 (138 mg, 0.22 mmol, 1 Eq) in THF (12 mL), TBAF was added at room temperature (2 mL, 1 M in THF). The resulting mixture was stirred at room temperature overnight. The reaction mixture was diluted with water (30 mL) and extracted with ethyl acetate (30 mL). The aqueous phase was separated and extracted with ethyl acetate (3 × 30 mL). The organic phases were combined and washed with water (2 × 30 mL) and brine (2 × 30 mL), followed by drying with sodium sulfate. The solvent was removed under reduced pressure. The residue was purified by silica gel flash column chromatography with dichloromethane and methanol elution (20:1) to furnish compound **1b** as a viscous oil (70 mg, 81% yield). ^1^H-NMR (700 MHz, CDCl_3_): δ 3.77–3.79 (m, 1 H), 3.67–3.72 (m, 1 H), 3.59–3.64 (m, 2 H), 3.35–3.42 (m, 1 H), 3.27–3.31 (m, 1 H), 3.24–3.26 (m, 1 H), 2.58 (dd, *J* = 19.6, 5.6 Hz, 1 H), 2.19 (d, *J* = 19.6 Hz, 1 H), 1.81–1.91 (m, 3 H), 1.71–1.79 (m, 2 H), 1.58–1.66 (m, 2 H), 1.45–1.58 (m, 6 H), 1.35–1.43 (m, 5 H), 1.02–1.31 (m, 8 H), 0.92–0.98 (m, 1 H), 0.90 (s, 3 H), 0.61 (s, 3 H). ^13^C-NMR (175 MHz, CDCl_3_), δ 77.69, 70.32, 69.10, 68.63, 63.16, 55.49, 54.76, 40.55, 38.96, 37.75, 34.93, 34.02, 33.84, 29.01, 25.64, 24.92, 24.84, 24.73, 24.70, 22.59, 21.83, 19.03, 17.51, 11.16. MS for C_24_H_42_O_4_ calculated: *m*/*z* 417.3 (M + Na)^+^; found: 417.3.

### 3.2. Biological Activity-Luciferase Reporter Gene Assays and Survival/Proliferation Assay

Cell culture. HEK293T cells (ATCC, Manassas, VA, USA) were constructed to the TGR5-overexpressing HEK293T cells in our team as previously reported [38]. The cells were cultured in high-glucose Dulbecco’s modified Eagle’s medium (DMEM; Thermo Fisher, Pittsburg, PA, USA) with L-glutamine supplemented with 10% (vol/vol) heat-inactivated fetal bovine serum (Atlas Biologicals, Fort Collins, CO, USA) and 1% penicillin–streptomycin (Thermo Fisher). TGR5-overexpressing HEK293T cells were maintained in G418 (Thermo Fisher)-containing media until plating. Cells were seeded onto 24-well plates (5 × 10^5^ cells/well) 24 h before transfection.

Luciferase reporter gene assays. To evaluate TGR5 agonistic activity, HEK293T cells were transfected with human TGR5 expression vector, pCRE-Luc reporter vector, and pCMV-Renilla luciferase vector as an internal control for normalization of transfection efficiency. Plasmids were complexed with PEI (Promega, Madison, WI, USA) in OptiMEM (Invitrogen, Carlsbad, CA, USA). After 18 h, cells were treated with vehicle (DMSO) and the appropriate ligand as indicated (5 µM). Luciferase activities were assayed after 6 h using Luciferase Assay System (Promega) and an MLX luminometer (Dynex Technologies, Chantilly, VA, USA).

Effective Concentrations (EC_50_) of 50% and Efficacy Determination. Assays were performed by the above assay. To evaluate TGR5 activity of compounds, cells were transfected with 100 ng pCRE-luc reporter, along with pCMV-Renilla (10 ng) as an internal control, for normalization of transfection efficiency. Plasmids were complexed with 2 mL of OptiMEM (Invitrogen, Carlsbad, CA, USA), and cells were transfected for 18 h. The following day, cells were treated with vehicle and compound with increasing concentrations of 0.01, 0.1, 1.0, 10, and 100 µM. Luciferase was assayed 6 h later using Luciferase Assay System (Promega) reagents.

Survival/Proliferation Assay. Cell proliferation was determined by MTS assay, which was conducted with the Cell Titer 96 Aqueous Cell Proliferation Kit (Promega). To test IC_50_, 3×10^3^ HEK 293T cells cultured in a 96-well plate were treated with increasing concentrations of compounds **1a**–**1c** and LCA for 48 h. After adding the MTS reagents, the plate was incubated for 3 h in a humidified, 37 °C incubator supplemented with a 5% CO_2_ atmosphere. The plate was read with a 96-well spectrometer using a 490 nm filter. The half-maximal inhibitory concentration (IC_50_) was described as the drug concentration that induced viability decrease by 50%.

### 3.3. Computational Modeling

The TGR5 protein structure was downloaded from the RCSB Protein Data Bank (PDB id 7cfm [55]). We used our in-house-developed all-around docking (AAD) [42,43] method to dock LCA and its 3 analogs **1a**–**1c** onto the whole surface of the TGR5 protein to automatically search for the best binding pocket and docking pose of each compound. The protein interaction analysis was performed using our in-house-developed LiAn (Legion Interfaces Analysis) [56] program, which could estimate and display protein–ligand or protein–protein interactions (including hydrogen bond, salt–bridge, water–bridge, π–interactions, hydrophobic interactions, halogen bond, etc.) for single-protein structures or massive structures from molecular dynamics simulations. The protein structure figure was produced using PyMoL (The PyMoL Molecular Graphics System, Version 2.0 Schrödinger, LLC, San Diego, CA, USA). The two-dimensional interaction diagram was produced using Schrödinger Maestro software.

## 4. Conclusions

In short, we successfully characterized a novel strategy for the development of bioactivity fragments based on the endogenous bile acid LCA to modulate the activity of LCA-related receptor TGR5. We developed a feasible strategy to synthesize these fragments. Three novel analogs, **1a**–**1c**, and **LCA** were evaluated using luciferase reporter gene assays and MTS assays. The initial results indicated that compounds **1a** and **1b** improved the hydrophilicity of the fragments and displayed agonist activities for TGR5. Our computational modeling supported this result by showing that compounds **1a** and **1b** could form more effective hydrogen bonds with hydrophilic residues. To the best of our knowledge, these lead compounds have not been previously reported in the literature. We hypothesize that this method could be applied to modify other bile acids to potentially improve hydrophilicity. Currently, we are in the process of evaluating the biological activity of these novel structure fragments.

## Data Availability

Not applicable.
